# *In situ* hybridization of an MXene/TiO_2_/NiFeCo-layered double hydroxide composite for electrochemical and photoelectrochemical oxygen evolution†

**DOI:** 10.1039/c8ra02349b

**Published:** 2018-06-05

**Authors:** Ningxian Hao, Yang Wei, Jialiang Wang, Zhiwei Wang, Zhaohua Zhu, Shulin Zhao, Min Han, Xiao Huang

**Affiliations:** Institute of Advanced Materials (IAM), Nanjing Tech University (NanjingTech) 30 South Puzhu Road Nanjing 211816 P. R. China iamxhuang@njtech.edu.cn; Jiangsu Key Laboratory of Biofunctional Materials School of Chemistry and Materials Science, Nanjing Normal University 1 Wenyuan Road Nanjing 210023 P. R. China

## Abstract

Electrochemical and photoelectrochemical (PEC) oxygen evolution reactions (OER) are receiving considerable attention owing to their important roles in the overall water splitting reaction. In this contribution, ternary NiFeCo-layered double hydroxide (LDH) nanoplates were *in situ* hybridized with Ti_3_C_2_T_*x*_ (the MXene phase) *via* a simple solvothermal process during which Ti_3_C_2_T_*x*_ was partially oxidized to form anatase TiO_2_ nanoparticles. The obtained Ti_3_C_2_T_*x*_/TiO_2_/NiFeCo-LDH composite (denoted as TTL) showed a superb OER performance as compared with pristine NiFeCo-LDH and comercial IrO_2_ catalyst, achieving a current density of 10 mA cm^−2^ at a potential of 1.55 V *versus* a reversible hydrogen electrode (*vs.* RHE) in 0.1 M KOH. Importantly, the composite was further deposited on a standard BiVO_4_ film to construct a TTL/BiVO_4_ photoanode which showed a significantly enhanced photocurrent density of 2.25 mA cm^−2^ at 1.23 V *vs.* RHE under 100 mW cm^−2^ illumination. The excellent PEC-OER performance can be attributed to the presence of TiO_2_ nanoparticles which broadened the light adsorption to improve the generation of electron/hole pairs, while the ternary LDH nanoplates were efficient hole scavengers and the metallic Ti_3_C_2_T_*x*_ nanosheets were effective shuttles for transporting electrons/ions. Our *in situ* synthetic method provides a facile way to prepare multi-component catalysts for effective water oxidation and solar energy conversion.

## Abstract 2

An *in situ* prepared Ti_3_C_2_T_*x*_/TiO_2_/NiFeCo-LDH composite showed excellent performance in both electrochemical and photoelectrochemical oxygen evolution reactions.

## Graphical abstract

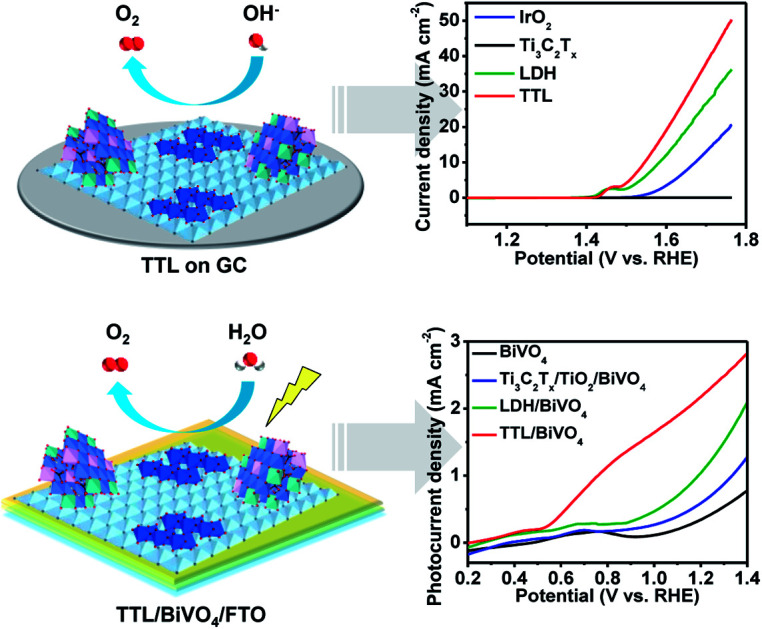

## 1. Introduction

Over the past years, considerable efforts have been devoted to the exploration of clean and renewable energy sources. The electrochemical and photoelectrochemical (PEC) splitting of water is highly anticipated for the sustainable production of hydrogen and/or oxygen.^1,2^ The oxygen evolution reaction (OER), as one of the two half reactions of water splitting, involving complex electron and ion transfer that usually leads to sluggish kinetics and poor energy conversion efficiency.^3,4^ Traditional precious-metal-based oxides such as RuO_2_ and IrO_2_ are among the most active electrocatalysts towards the OER.^5,6^ However, their scarcity and accompanying high cost have limited their mass production and wide application. Recently, transition-metal-based compounds, such as sulfides,^7,8^ (oxy)hydroxides,^9,10^ oxides^11–13^ and layered double hydroxides (LDHs)^14–17^ have shown great potential for OER. The transition metal LDHs, which consist of positively charged layers where divalent and trivalent metal cations (*e.g.*, Ni^2+^, Co^2+^, Mg^2+^, Al^3+^ and Fe^3+^) are coordinated to hydroxyl anions^14–16^ and charge-balancing anions (*e.g.*, CO_3_^2−^, Cl^−^, and/or SO_4_^2−^)^14,17^ inserted between adjacent layers, are particularly attractive, because of their versatility in both chemical composition and morphology as well as relatively low costs. Several works have indicated that compared to binary LDHs, ternary ones exhibited further improved OER performance.^18–20^ For example, introducing Fe to NiCo-LDH could boost its OER performance, most likely due to the enhanced conductivity and increased level of structural disorder.^9,18^ More importantly, many LDHs are found to be semiconductors with suitable bandgaps in the visible-light range (*e.g.*, Co_2_Fe-LDH, Ni_0.75_Fe_0.25_-LDH, CoAl-LDH, CuCr-LDH) and therefore show promises in PEC water splitting.^21–25^ However, LDHs generally suffer from poor carrier mobilities and aggregation during film forming,^26,27^ which hinder charge separation and transfer. Therefore, LDHs have been combined with conductive additives such as graphene-based materials and carbon nanotubes.^28–30^ Unfortunately, these conductive materials generally show poor surface hydrophilicity which may restrict easy access of aqueous electrolyte.

Recently, MXenes – a large family of layered materials have drawn considerable attention, which are commonly produced by the extraction of A from the ternary carbides or nitrides with a formula of M_*n*+1_AX_*n*_, where M is an early transition metal, A is an A-group element and X is C and/or N.^31–33^ As one of the most widely studied MXene, Ti_3_C_2_T_*x*_ (T_*x*_ represents the terminal groups such as –(OH)_*x*_ and –F_*x*_),^34^ has demonstrated outstanding performance in various electrochemical applications,^35,36^ thanks to its good conductivity and hydrophilicity, as well as high electronegativity.^37–39^ Importantly, previous studies found that Ti_3_C_2_T_*x*_ nanosheets/flakes could provide Ti source for the surface growth of TiO_2_ for solar energy harvesting.^40–42^ Therefore, combining Ti_3_C_2_T_*x*_/TiO_2_ and co-catalyst such as LDHs is expected to produce high performance PEC photocatalyst.

In this work, a composite of Ti_3_C_2_T_*x*_/TiO_2_/NiFeCo-LDH (denoted as TTL) was prepared by growth of NiFeCo-LDH nanoplates on surfaces of Ti_3_C_2_T_*x*_ nanosheets solvothermally, during which TiO_2_ nanoparticles were simultaneously formed. The hybrid material showed excellent electrocatalytic activity toward OER and achieved a current density of 10 mA cm^−2^ in 0.1 M KOH at 1.55 V *vs.* RHE, which is among the best reported OER catalysts. In addition, the composite material was combined with BiVO_4_ for PEC-OER and exhibited the much enhanced activity in comparison with the pristine BiVO_4_. The excellent performance for both OER and PEC-OER resulted from a synergistic effect from all its components.

## 2. Experimental section

### 2.1 Chemicals

Titanium aluminum carbide (Ti_3_AlC_2_, ≥98%) was purchased from Tianmazhihui Technology Company (Beijing, China). Potassium hydroxide (KOH, 85%), hydrogen fluoride (HF, 40%) and Ni(ii) chloride hexahydrate (99.9%) were purchased from J&K Chemical (Beijing, China). 1,4-Naphthalene dicarboxylic acid (1,4-H_2_NDC, 95%) was purchased from Aladdin (Shanghai, China). *N*,*N*-Dimethylformamide (DMF, 99.5%) and ethanol (99.7%) were purchased from Shanghai Chemical Reagent CO., LTD (Shanghai, China). Iridium oxide (IrO_2_, 99.9%), Nafion and Co(ii) chloride hexahydrate (99.9%) were purchased from Sigma-Aldrich (Beijing, China). Iron(iii) chloride hexahydrate (97%) was purchased from Stream Chemicals, INC. (Newburyport, USA). *N*,*N*-Dimethyltetradecylamine (90%) was purchased from Tokyo Chemical Industry CO., LTD. (Tokyo, Japan). *n*-Butanol (99%) was purchased from Shanghai Shenbo Chemical CO., LTD. (Shanghai, China). BiVO_4_ coated fluorine-doped SnO_2_ (FTO) glass substrates with a BiVO_4_ mass loading of 0.5 mg cm^−2^ were purchased from TOEI Technology (Hangzhou, China). The deionized (DI) water was purified using a Milli-Q3 System (Millipore, France). All chemicals were used without further purification.

### 2.2 Preparation of Ti_3_C_2_T_*x*_ nanosheets

5 mg of Ti_3_AlC_2_ powder was mixed with 30 mL of ethanol and ball-milled to form a homogeneous slurry. The slurry was then centrifuged and dried at 60 °C for 12 h before being ground into a fine powder. Then, 180 mg of this powder, 600 mg of 1, 4-H_2_NDC, 3 mL of 40% HF, and 60 mL of DI water were mixed in a 150 mL Teflon, heated and maintained at 180 °C for 6 h. After being cooled down to room temperature, the collected precipitate was washed with DMF for several times, and then sonicated in DMF for 16 h to yield isolated Ti_3_C_2_T_*x*_ nanosheets.

### 2.3 Preparation of NiFeCo-LDH nanoplates

NiFeCo-LDH nanoplates were synthesized using a solvothermal method in a reverse microemulsion system based on a previous report.^42^ Typically, 1.65 mL of *n*-butanol and 2.65 mL of *N*,*N*-dimethyltetradecylamine were mixed under sonication to form a reverse emulsion solution, into which, 450 μL of NiCl_2_ aqueous solution (0.2 mol L^−1^), 150 μL of FeCl_3_ aqueous solution (0.2 mol L^−1^), and 75 μL of CoCl_2_ aqueous solution (0.2 mol L^−1^) were added. The resulting mixture was then heated and maintained at 120 °C for 12 h in a 5 mL Teflon. After being cooled down to room temperature, the product was collected by centrifugation and washed with ethanol for at least 5 times.

### 2.4 Preparation of Ti_3_C_2_T_*x*_/TiO_2_/NiFeCo-LDH composite

The preparation method of Ti_3_C_2_T_*x*_/TiO_2_/NiFeCo-LDH was similar to that of NiFeCo-LDH nanoplates, except that 4 mg of Ti_3_C_2_T_*x*_ nanosheets were mixed with the aforementioned growth solution for NiFeCo-LDH and then vibrated at 850 rpm in an oscillator for 2 h before being transferred to a 5 mL Teflon and heated at 120 °C for 12 h.

### 2.5 Characterizations

X-ray diffraction (XRD, SmartLab Rigaku) was performed with Cu Kα radiation (*λ* = 1.54 Å) as the X-ray source. Scanning electron microscopy (SEM, Hitachi S-4800) was used for the morphological analysis. To gain the microstructure and composition information, transmission electron microscopy (TEM, Hitachi HT7700), high resolution transmission electron microscopy (HRTEM, JEOL 2100F), and energy-dispersive X-ray (EDX) spectroscopy analyses were performed. The elemental analysis and oxidation state study of the samples were carried out by X-ray photoelectron spectroscopy (XPS, PHI 5000 VersaProbe), and the binding energies were corrected for specimen charging effects using the C 1s level at 284.6 eV as the reference.

### 2.6 Electrochemical measurements

Typically, 2.5 mg of the active material, *i.e.* TTL, NiFeCo-LDH, Ti_3_C_2_T_*x*_ or IrO_2_, was mixed with 400 μL deionized water, 100 μL ethanol and 10 μL Nafion, followed by sonication to form a uniform dispersion. Then, 3 μL of such dispersion was drop-casted onto the surface of a pre-polished glassy carbon (GC) electrode (with a diameter of 3 mm) and then dried naturally at room temperature overnight. Cyclic voltammetry (CV) and linear sweep voltammetry (LSV) measurements were conducted in a three-electrode system on an electrochemical station (Autolab 302N). A Pt foil was used as the counter electrode, a 3 M Ag/AgCl electrode as the reference electrode, and the catalyst-modified GC rotating disk electrode (RDE) as the working electrode. All measured potentials *versus* Ag/AgCl were converted to the reversible hydrogen electrode (RHE) based on the Nernst equation below:^45^

1 *E* (*vs*. RHE) = *E* (*vs*. Ag/AgCl) + 0.059 × pH + *E*_Ag/AgCl_

where *E* (*vs.* RHE) is the potential referred to RHE, *E* (*vs.* Ag/AgCl) is the applied potential against 3 M Ag/AgCl reference electrode, and *E*_Ag/AgCl_ is the standard potential of Ag/AgCl reference electrode. All the measurements were conducted in O_2_-saturated 0.1 M KOH solution (pH = 13). The CV and LSV curves were measured at scan rate of 100 mV s^−1^ and 2 mV s^−1^, respectively, at a rotating speed of 1600 rpm. Electrochemical impedance spectroscopy (EIS) measurements were conducted at a fixed potential of 1.57 V (*vs.* RHE) by applying an AC voltage with the amplitude of 5 mV over a frequency range of 100 kHz to 0.01 Hz. The electrical double-layer capacitance (*C*_dl_) of the catalyst was measured from the double-layer charging curves using CV in a small potential range of 1.411–1.464 V (*vs.* RHE) without apparent faradaic processes occurring (Fig. S7†). The plot of the current density difference at 1.438 V (*vs.* RHE) against the scan rate (20, 40, 60, 80, 100 mV s^−1^) was linearly fitted, and its slope was the *C*_dl_ of the tested catalyst (Fig. 4c).

The rotating ring-disk electrode (RRDE) measurements were carried out in a three-electrode cell (CHI 700e, Shanghai, China) using a Pt foil as the counter electrode, a 3 M Ag/AgCl electrode as the reference electrode, and a catalyst-modified RRDE (Garmy RDE710, Beijing, China) as the working electrode with a rotating speed of 1600 rpm in O_2_-saturated 0.1 M KOH solution. The RRDE includes a glassy carbon disk with a diameter of 5 mm and an area of 0.1963 cm^2^, and a Pt ring with an area of 0.1859 cm^2^.

### 2.7 Photoelectrochemical measurements

All PEC-OER tests were conducted on an electrochemical workstation (CHI 660E, CH Instruments, Shanghai) in a three-electrode cell using a 3 M Ag/AgCl standard electrode as the reference and a Pt foil (1.5 cm × 1.5 cm) as the counter electrode. Typically, 2.5 mg active material such as LDH, Ti_3_C_2_T_*x*_/TiO_2_ or TTL composite was mixed with 800 μL deionized water, 200 μL ethanol and 20 μL Nafion, followed by sonication to form a uniform dispersion. The concentration of Ti, Ni, Fe and Co elements in LDH, Ti_3_C_2_T_*x*_/TiO_2_ and TTL dispersions were measured by inductively coupled plasma optical emission spectrometer (ICP-OES), respectively (Table S1†). For fabrication of a typical photoanode, the loading of TTL onto the 1 cm × 1 cm BiVO_4_/FTO substrate was 175 μg by drop-wise casting 70 μL of 2.5 mg mL^−1^ dispersion, which contained ∼142 μg LDH and ∼33 μg Ti_3_C_2_T_*x*_/TiO_2_. In our control experiments, the loading of LDH on BiVO_4_/FTO was then ensured to be ∼142 μg (*i.e.* 57 μL of 2.5 mg mL^−1^ dispersion) and that of Ti_3_C_2_T_*x*_/TiO_2_ was ∼33 μg (*i.e.* 13 μL of 2.5 mg mL^−1^ dispersion). After the dispersion was drop-casted onto BiVO_4_/FTO, the photoanode was dried naturally at room temperature overnight.

Fluorine-doped SnO_2_ (FTO) glass substrates with surface deposited BiVO_4_ (BiVO_4_/FTO, purchased from TOEI, Hangzhou, China) and TTL/BiVO_4_/FTO (both with a film area of 1 cm × 1 cm) acted as the photoanode. All measurements were performed at room temperature in 0.5 M potassium phosphate (pH = 7) solution. A 300 W xenon lamp (PLX-SEX300, PerfectLight, Beijing) was used to irradiate the photoanode from the back at an intensity of 100 mW cm^−2^ determined by a power meter (PL-MW2000, PerfectLight, Beijing). The photo energy conversion efficiency (*η*) of the photoanodes was calculated by the LSV curves using the following equation:^46,47^

2 *η* = [1.23 − *E* (*vs.* RHE) × *J*/*P*_light_] × 100%

where 1.23 V is the equilibrium potential for OER, *E* (*vs.* RHE) is the applied potential *vs.* the RHE, *J* is the photocurrent density at the measured potential, and *P*_light_ (100 mW cm^−2^) is the power density of illumination.

EIS measurements of the photoanodes under illumination were performed on the electrochemical workstation with a 5 mV amplitude perturbation between 100 kHz and 0.01 Hz at open circuit potential.

The incident photon-to-current efficiency (IPCE) was calculated using the equation below:^46^

3 IPCE = [[1240 × (*J*_light_ − *J*_dark_)]/(*P* × *λ*)] × 100%

where *J*_light_ and *J*_dark_ are the measured photocurrent and dark current density (mA cm^−2^) obtained at 1.23 V *vs.* RHE, respectively. *P* is the measured irradiance at a specific wavelength (mW cm^−2^), and *λ* is the wavelength of the incident light (nm).

## 3. Results and discussion

### 3.1 Preparation and characterization of Ti_3_C_2_T_*x*_/TiO_2_/NiFeCo-LDH composite

**Fig. 1:**
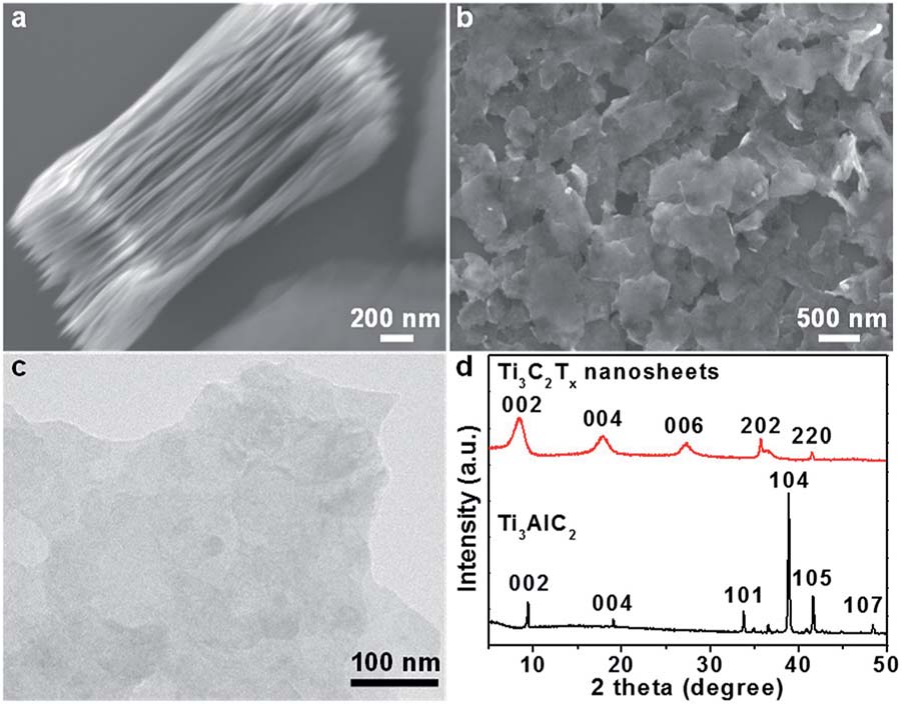
(a) SEM image of layered Ti_3_C_2_T_*x*_. (b) SEM and (c) TEM images of isolated Ti_3_C_2_T_*x*_ nanosheets after sonication. (d) XRD patterns of Ti_3_C_2_T_*x*_ nanosheets and Ti_3_AlC_2_ bulk crystals.

In a typical process, Ti_3_C_2_T_*x*_ nanosheets were firstly prepared by selectively removing Al layers in bulk Ti_3_AlC_2_ crystals with HF to obtain the layered structures as shown in the scanning electron microscopy (SEM) image in Fig. 1a, followed by sonication to produce exfoliated Ti_3_C_2_T_*x*_ nanosheets (Fig. 1b and c), and a typical nanosheet showed a thickness of ∼6 nm (Fig. S1†). The Ti_3_C_2_T_*x*_ nanosheets and Ti_3_AlC_2_ were further characterized by X-ray diffraction (XRD, Fig. 1d). The diffraction peak at 39°, which corresponds to the (104) planes of Ti_3_AlC_2_,^48^ is not observed in the pattern of Ti_3_C_2_T_*x*_, suggesting the complete removal of Al layers. In addition, the (002) peak of Ti_3_C_2_T_*x*_ compared to that of Ti_3_AlC_2_ shifted from 9.3° to 8.6° and became broadened, indicating an enlarged interlayer spacing (from 0.94 nm to 1 nm) and possibly a reduced thickness. The chemical composition and oxidization states of the as-exfoliated Ti_3_C_2_T_*x*_ nanosheets were studied by X-ray photoelectron spectroscopy (XPS) (Fig. S2†), and surface-terminated groups like –(OH)_*x*_ and –F_*x*_ were identified,^49,50^ which rendered the Ti_3_C_2_T_*x*_ nanosheets hydrophilic and negatively charge.

**Scheme 1:**
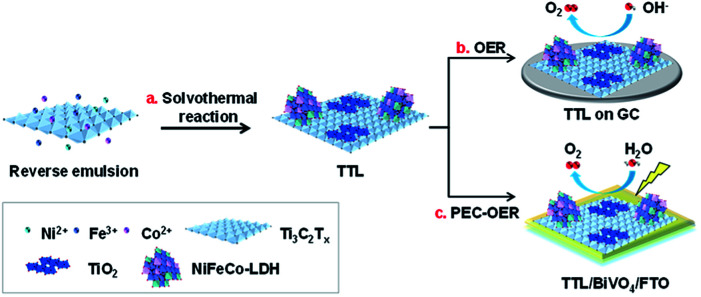
Illustration of the preparation of TTL for electrochemical and photoelectrochemical oxygen evolution reactions.

**Fig. 2:**
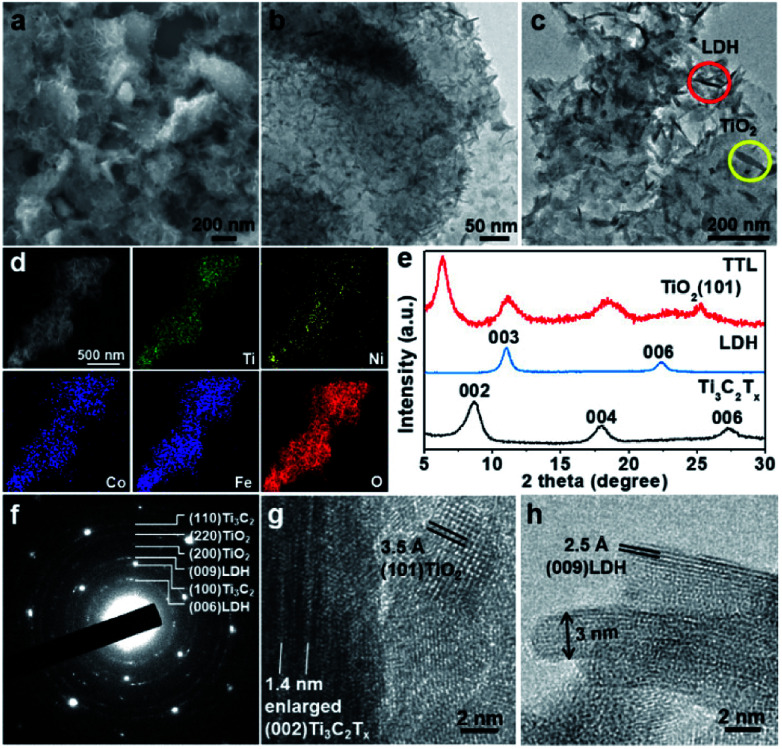
(a) SEM image of TTL. (b), (c) TEM images of the TTL. Inset: The LDH nanosheets (red circle) and TiO_2_ nanospindles (yellow circle). (d) STEM image and EDX mapping of TTL. (e) XRD patterns of TTL, LDH and Ti_3_C_2_T_*x*_ nanosheets. (f) Selected area electron diffraction of TTL. (g) HRTEM image of TiO_2_ and folded Ti_3_C_2_T_*x*_ edge. (h) A side-view HRTEM image of LDH nanoplates.

The as-prepared Ti_3_C_2_T_*x*_ nanosheets were then used as synthetic templates for the growth of NiFeCo-LDH *via* a solvothermal reaction as illustrated in Scheme 1a. The synthetic process began with mixing Ni^2+^, Fe^3+^ and Co^2+^ precursors as well as Ti_3_C_2_T_*x*_ in a reverse microemulsion system,^44,51^ in which *N*,*N*-dimethyltetradecylamine and *n*-butanol were mixed with a volume ratio of 8 : 5. The surface-terminated groups (*i.e.* –(OH)_*x*_ and –F_*x*_) of Ti_3_C_2_T_*x*_ nanosheets might absorb Ni^2+^, Fe^3+^, and Co^2+^ ions *via* the electrostatic interaction. After the mixture was heated at 120 °C in an autoclave for 12 h, NiFeCo-LDH nanoplates together with TiO_2_ nanoparticles were formed on the surfaces of Ti_3_C_2_T_*x*_ nanosheets (Fig. 2a–c). Importantly, different from the NiFeCo-LDH nanoplates synthesized directly in solution and deposited randomly on a surface (*e.g.* on a copper grid) as shown in Fig. S3,† the NiFeCo-LDH nanoplates tend to stand up on the surfaces of Ti_3_C_2_T_*x*_ with edges largely exposed. Differently, TiO_2_ nanoparticles show spindle-like morphology (Fig. 2c). Our control experiment result indicated that Ti_3_C_2_T_*x*_ nanosheets which underwent a similar solvothermal treatment also showed surface deposited TiO_2_ nanospindles (Fig. S4†). It has been reported previously that surface defects might form on Ti_3_C_2_T_*x*_ nanosheets as a result of HF treatment, which provided preferential nucleation sites and Ti source for the growth of TiO_2_ in presence of air at elevated temperatures.^41,52,53^ Moreover, EDX mapping of a typical hybrid material in Fig. 2d shows the uniform distribution of Ti, Ni, Co, Fe and O elements, further indicating the successful preparation of the composite. The EDX spectrum in Fig. S5a† shows that the atomic ratio of Ni : Fe : Co in LDH was about 3 : 1 : 0.5, and Cl element was also detected. In addition, from the FT-IR spectrum of LDH (Fig. S5b†), a strong vibration of CO_3_^2−^ at ∼1377 cm^−1^ was observed.^56,57^ These imply that both Cl^−^ and CO_3_^2−^ ions may act as the charge-balancing anions in the LDH nanoplates. The crystal structure of the composite was analyzed by XRD and compared with pristine Ti_3_C_2_T_*x*_ and LDH (Fig. 2e). The observed diffraction peak at 11.1° can be assigned to the (003) planes of NiFeCo-LDH (JCPDS no. 51-0463) based on previous reports as well as our control experiment (Fig. S6†).^54,55^ The peak present at 25.2° can be assigned to the (101) planes of TiO_2_ with the anatase phase (JCPDS no. 21-1272).^58,59^ It is interesting to note that the peak for the (002) planes of Ti_3_C_2_T_*x*_ shifts from 8.6° to 6.3° after hybridization, indicating an increased interlayer spacing (from 1 nm to 1.4 nm). This can be attributed to the intercalation of metal ions and surface oxidation of Ti_3_C_2_T_*x*_ during the solvothermal process. Selected area electron diffraction (SAED) and high resolution transmission electron microscopy (HRTEM) characterization were further applied to study the microstructure of the composite. As shown in Fig. 2f, the SAED pattern on a typical hybrid material lying flatly on a copper grid clearly shows the (100) and (110) spots for Ti_3_C_2_T_*x*_ with the six-fold symmetry. Besides, discontinued rings for (200) TiO_2_, (220) TiO_2_, (006) LDH and (009) LDH planes are also observed. Fig. 2g shows a typical square lattice pattern of anatase TiO_2_ along the [001] zone axis, with a lattice spacing of 3.5 Å for (101) planes. On the same image, a folded Ti_3_C_2_T_*x*_ could also be observed which shows enlarged interlayer spacing of ∼1.4 nm, consistent with the XRD peak at ∼6.3° (Fig. 2e). A side-view HRTEM image of NiFeCo-LDH nanoplates reveals a lattice spacing of 2.5 Å for the (009) planes as shown in Fig. 2h. From this image, the thickness of the LDH nanoplates can be estimated to be ∼3 nm.

**Fig. 3:**
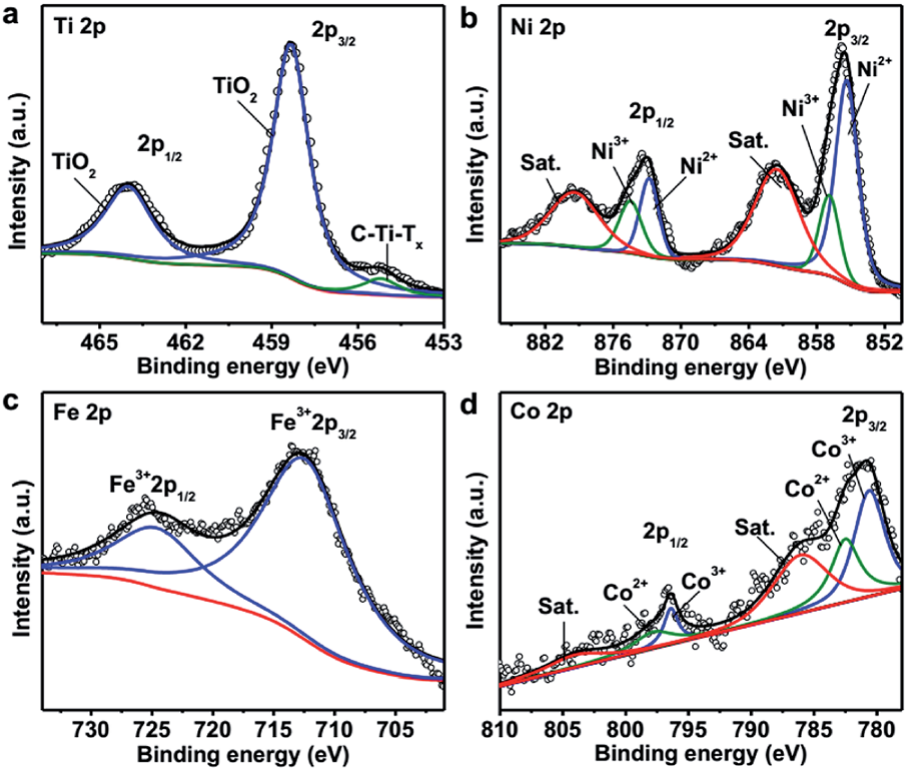
XPS (a) Ti 2p, (b) Ni 2p, (c) Fe 2p and (d) Co 2p spectra of TTL.

The composition and oxidization states of the hybrid material were further analyzed with XPS and shown in Fig. 3. The Ti 2p spectrum (Fig. 3a) shows a doublet at binding energies of 458.3 eV and 464 eV, which can be assigned to TiO_2_. The other peak at 455.2 eV is attributable to C–Ti–T_*x*_ (T is O, OH or F).^60–62^ It is important to note that compared with the Ti 2p spectrum of Ti_3_C_2_T_*x*_ nanosheets before hybridization (Fig. S1a†), the amount of oxide greatly increased after the solvothermal process. The Ni 2p spectrum shows two sets of doublets for Ni^2+^ (855.4 eV and 872.8 eV) and Ni^3+^ (857 eV and 874.5 eV), along with two satellite peaks (denoted as Sat.) at 861.5 eV and 879.4 eV, respectively (Fig. 3b).^63–65^ For the Fe 2p spectrum, the binding energies of 712.4 eV and 724.6 eV are corresponding to Fe 2p_3/2_ and Fe 2p_1/2_ bands of Fe^3+^, respectively (Fig. 3c).^66^ Similarly, the deconvolution of the Co 2p spectrum suggests the presence of Co^2+^ (782.5 eV and 797.8 eV) and Co^3+^ (780.6 eV and 796.4 eV) species, along with two satellite peaks at 786.1 eV and 804 eV, respectively (Fig. 3d).^63,65^ As a result, Co and Ni exist as multiple valence state and Fe provides +3 species in the hybrid material which are consistent with the pure LDH (Fig. S7†). Based on the XPS and ICP-OES results, the concentrations of the various components in TTL were calculated as shown in Tables S1–S3.†

### 3.2 Electrochemical measurements

**Fig. 4:**
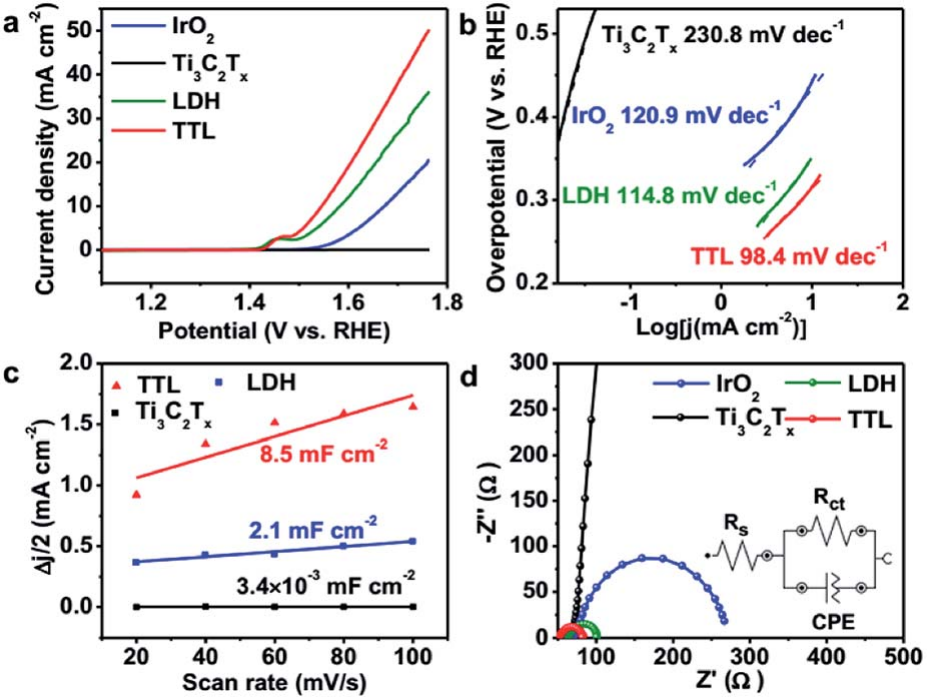
(a) LSV curves for electrodes modified by TTL, LDH, IrO_2_ and Ti_3_C_2_T_*x*_ for OER at the scan rate of 2 mV s^−1^. (b) The corresponding Tafel plots. (c) Current density difference at 1.438 V (*vs.* RHE) plotted against scan rate to give the double-layer capacitance (*C*_dl_). (d) Nyquist plots for the various electrodes measured at a potential of 1.57 V (*vs.* RHE). The inset is the equivalent circuit. *R*_ct_ is the charge transfer resistance, *R*_s_ is solution resistance, and CPE is the constant phase element.

We first examined the electrocatalytic activity of the TTL composite toward OER in O_2_-saturated 0.1 M KOH solution. For comparison, the performance of NiFeCo-LDH, Ti_3_C_2_T_*x*_, and commercial IrO_2_ towards OER was also tested on the basis of equal loading of active catalysts (*i.e.* 0.21 mg cm^−2^). Fig. 4a shows the linear-sweep voltammetry (LSV) measurements of the various catalysts deposited on glassy carbon (GC) electrode. The TTL modified electrode showed an oxidation peak at 1.4–1.5 V *vs.* RHE, which can be assigned to the Ni^2+/3+^ to Ni^3+/4+^ as well as the Co^2+/3+^ to Co^3+/4+^ redox processes,^67,68^ consistent with their corresponding cyclic voltammetry (CV) curves (Fig. S8†). Above this potential, current density rises sharply with O_2_ evolution.^67,69–71^ In addition, the TTL hybrid catalyst achieved a current density of 10 mA cm^−2^ at a potential of 1.55 V *vs.* RHE, which outperforms the state-of-the-art IrO_2_ catalyst (1.67 V *vs.* RHE) and is comparable among the currently best OER catalysts under the same measurement conditions (Table S4†). Furthermore, the Tafel slope for the TTL composite catalyst (98.4 mV dec^−1^) was lower than that of NiFeCo-LDH (114.8 mV dec^−1^), IrO_2_ (120.9 mV dec^−1^), and Ti_3_C_2_T_*x*_ (230.8 mV dec^−1^) (Fig. 4b), suggesting a more favored OER kinetics of the TTL hybrid catalysts.

The much enhanced performance of TTL catalyst compared to their individual components can be attributed to the synergistic coupling effect. The effective surface areas for the various electrocatalysts for OER were first analyzed based on the double-layer capacitance (C_dl_), which was measured by CV with different scan rates over 1.411–1.464 V *vs.* RHE, that is, a potential range with no apparent faradaic processes (Fig. S9†). Fig. 4c shows the current density plotted against scan rate at a potential of 1.438 V *vs.* RHE, from which *C*_dl_ can be calculated. As expected, the as exfoliated Ti_3_C_2_T_*x*_ nanosheets showed a low *C*_dl_ of 3.4 × 10^−3^ mF cm^−2^, whereas NiFeCo-LDH nanoplates showed a slightly larger *C*_dl_ of 2.1 mF cm^−2^, and compared to the LDH, the TTL composite exhibited a more than 4 times increased *C*_dl_ of 8.5 mF cm^−2^, suggesting that the Ti_3_C_2_T_*x*_ nanosheets not only supported the NiFeCo-LDH nanoplates but also facilitated the easy access of aqueous electrolyte to the active surfaces of LDH nanoplates.^28^ Furthermore, the metal-like Ti_3_C_2_T_*x*_ nanosheets could enable fast charge/ion transport within the hybrid catalyst film, as indicated by the electrochemical impedance spectroscopy (EIS) measurement results (Fig. 4d). In addition, rotating ring-disk electrode (RRDE) measurement was carried out on the TTL-based electrode. A much smaller current was measured on the ring electrode compared to that on the disk electrode (Fig. S10†), suggesting a four-electron pathway for water oxidation (4OH^−^ → O_2_ + 2H_2_O + 4e^−^) with negligible formation of peroxide intermediate.^50,72^ The Nyquist plots of the various electrodes were fitted by the RC circuit model as shown in the inset of Fig. 4d, which includes a solution resistance (*R*_s_), a charge transfer resistance (*R*_ct_) and a constant phase component (CPE). The obtained *R*_ct_ values are listed in Table S5†. As consistent with the LSV curves and Tafel slopes, the *R*_ct_ of TTL is smaller than NiFeCo-LDH and IrO_2_, demonstrating a faster charge transfer during OER.

### 3.3 Photoelectrochemical measurements

**Fig. 5:**
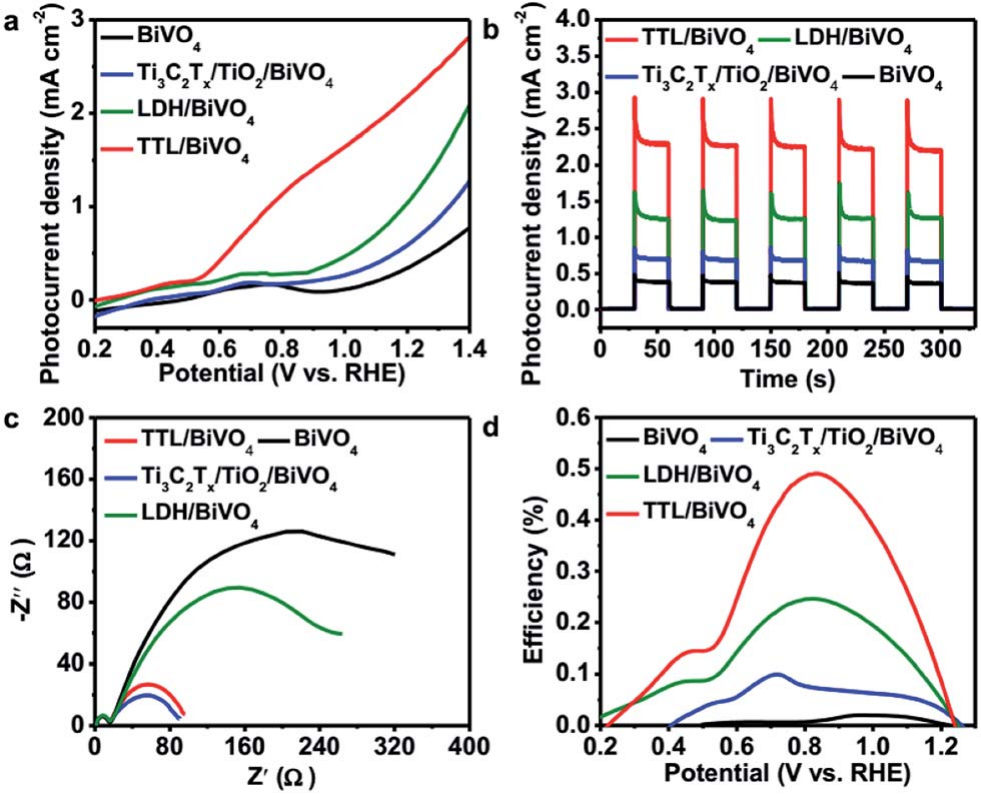
(a) Photocurrent–potential curves of pristine BiVO_4_ and TTL/BiVO_4_ photoanodes measured under 100 mW cm^−2^ illumination. (b) Chronoamperometry curves of BiVO_4_ and TTL/BiVO_4_ photoanodes performed under on–off illumination cycles at a potential of 1.23 V (*vs.* RHE). (c) EIS plots of BiVO_4_ and TTL/BiVO_4_ measured under illumination at open circuit potential over a frequency range from 100 kHz to 0.01 Hz. (d) Applied bias photo-to-current conversion efficiency (ABPE) curves of pristine BiVO_4_ and TTL/BiVO_4_ photoanodes derived from (a). All measurements were conducted in 0.5 M potassium phosphate solution (pH = 7).

Considering the fact that TiO_2_ is a typical semiconductor for light harvesting^46,73^ and transition metal LDHs have been known as promising co-catalysts or hole scavengers for PEC-OER,^46,74^ the simultaneous formation of TiO_2_ and NiFeCo-LDH on Ti_3_C_2_T_*x*_ is expected to generate an efficient hybrid catalyst for PEC-OER. Therefore, we used BiVO_4_ as a model PEC-OER catalyst to study the performance of TTL composite by casting it on pristine BiVO_4_/FTO as a photoanode. The PEC-OER tests were performed in 0.5 M potassium phosphate buffer (pH = 7). As shown in the photocurrent–potential curves in Fig. 5a, under 100 mW cm^−2^ illumination, the TTL/BiVO_4_ photoanode showed a much higher photocurrent density compared with the pristine BiVO_4_ over a potential window of 0.2 to 1.4 V (*vs.* RHE), and achieved a current density of 2.25 mA cm^−2^ at 1.23 V (*vs.* RHE), which is about 5 times higher than that of the pristine BiVO_4_ (0.39 mA cm^−2^). This also outperforms some previously reported LDH-based hybrids like N-deficient C_3_N_4_/N-doped graphene/NiFe-LDH hybrid and reduced titania@LDH hybrid.^60,75^ Additionally, the onset potential (potential at which photocurrent exceeds to 0.02 mA cm^−2^)^76^ shifted from 0.51 V for the pristine BiVO_4_ to 0.25 V *vs.* RHE, indicating the enhanced PEC-OER performance in TTL/BiVO_4_ photoanode. To further study the photoresponse of TTL/BiVO_4_ in comparison with pristine BiVO_4_, chronoamperometry measurements were carried out at 1.23 V (*vs.* RHE) under on–off illumination cycles (Fig. 5b). The current density of TTL/BiVO_4_ is at least 5 times higher than that of the pristine BiVO_4_, in addition to a prompt and steady response over consequent cycles. However, a spike was observed at the beginning of each illumination cycle, and quickly reduced to a steady state plateau, which can be attributed to a sudden generation of charge carriers and partial recombination.^21,77^ This can be due to the fact the TTL was drop-casted onto pristine BiVO_4_, which might result in poorer interface compared to those directly grown on BiVO_4_ as reported previously.^33,43,78^ To explore the charge transport properties of the PEC catalysts, EIS analysis was conducted at open circuit potential. From the obtained Nyquist plots in Fig. 5c, it can be seen that the semicircle for TTL/BiVO_4_ photoanode under illumination is smaller than that of the pristine BiVO_4_ photoanode, pointing to a much improved charge transfer between the anode and electrolyte, which is in line with the trend observed in Fig. 5a. Furthermore, the applied bias photon-to-current efficiency (ABPE) of pristine BiVO_4_ and TTL/BiVO_4_ photoanodes was plotted as a function of applied potential *vs.* RHE (Fig. 5d), which was derived from the LSV curves in Fig. 5a. It is obvious that the photoconversion efficiency of TTL/BiVO_4_ is much higher than that of the pristine BiVO_4_ photoanode over the bias window of 0.2–1.23 V *vs.* RHE. The maximum efficiency of TTL/BiVO_4_ is 0.5% (at 0.84 V *vs.* RHE) which is over 20 times higher than that of pristine BiVO_4_ photoanode (0.02% at 1.0 V *vs.* RHE). We further measured the incident photon-to-current efficiency (IPCE) of the various photoanodes at a constant applied potential of 1.23 V *vs.* RHE under monochromatic irradiation from 350 nm to 600 nm (Fig. S11†). All the hybrid photoanodes demonstrated much higher IPCEs than the pristine BiVO_4_ photoanode. The TTL/BiVO_4_ achieved the highest performance with a maximum efficiency of 44.6% at 380 nm. It is worth noting that the efficiency for both the Ti_3_C_2_T_*x*_/TiO_2_/BiVO_4_ and TTL/BiVO_4_ photoanodes peaked at ∼380 nm, which agreed with the fact that TiO_2_ with a band-gap of ∼3.2 eV acted as a UV responsive photocatalyst.

**Fig. 6:**
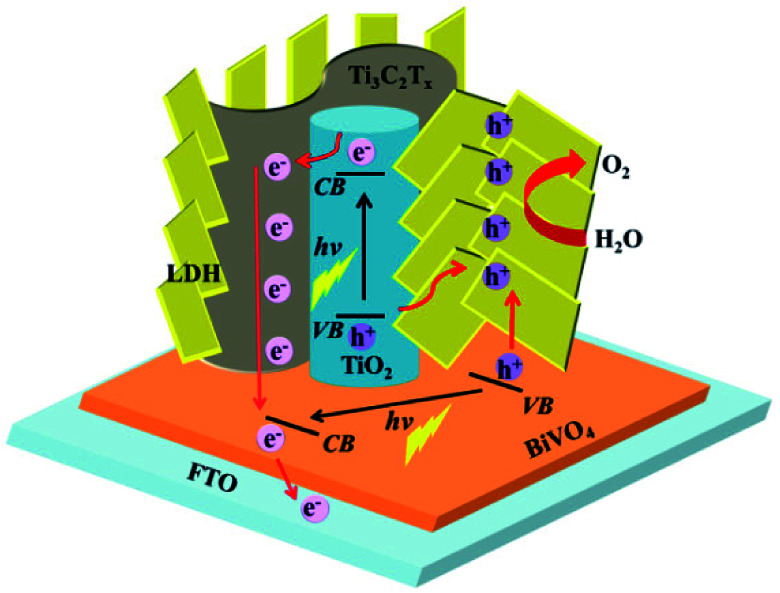
Schematic illustration of the proposed charge-transfer pathways in TTL/BiVO_4_ photoanode.

The possible charge-transfer pathways in the TTL/BiVO_4_ photoanode during PEC-OER process was proposed as shown in Fig. 6. The much improved PEC performance of TTL/BiVO_4_ photoanode can be attributed to the following reasons. First, the anatase TiO_2_ nanoparticles with a wide bandgap of ∼3.2 eV absorbed light mostly in the UV region (Fig. S12†),^25,43,79^ which supplement the absorption of BiVO_4_ with a relatively narrow bandgap (∼2.4 eV).^78–80^ This was also evidenced by the IPCE results (Fig. S11†). Besides, LDHs have also shown weak semiconducting properties for light harvesting.^21,23–25,79^ Second, the conductive and hydrophilic Ti_3_C_2_T_*x*_ nanosheets might act as effective shuttles for electron/ion transport, which was also reflected in the EIS analysis results (Fig. 5c). Most importantly, considering the fact that the valence band levels of most CoFe or NiFe-based LDHs (about −5 to −6 eV)^21,46,81^ are higher than that of TiO_2_ (about −7.2 eV)^44^ and BiVO_4_ (about −7.1 eV)^82,83^ (all the band level are related to the vacuum level), holes generated in BiVO_4_ or TiO_2_ upon light irradiation could be effectively scavenged by the LDH nanoplates for the oxidation reactions of Ni^2+/3+^ to Ni^3+/4+^ as well as the Co^2+/3+^ to Co^3+/4+^ to take place.

## 4. Conclusions

In summary, exfoliated Ti_3_C_2_T_*x*_ nanosheets were used as synthetic templates for the growth of NiFeCo-LDH nanoplates, and also provided Ti source for the formation of anatase TiO_2_ nanoparticles. The resulting composite showed excellent performance in OER. The NiFeCo-LDH nanoplates were mostly standing up on Ti_3_C_2_T_*x*_ with their ∼3 nm thick edges largely exposed, leading to a high active surface area for redox reactions. In addition, the Ti_3_C_2_T_*x*_ nanosheets were highly conductive and hydrophilic, allowing for the easy access of electrolyte and transport of electrons/ions. When the composite was further combined with the standard BiVO_4_ film, excellent performance in PEC-OER was also achieved. In addition to the high catalytic activity of NiFeCo-LDH nanoplates and conductivity of Ti_3_C_2_T_*x*_, TiO_2_ nanoparticles which were uniformly distributed on Ti_3_C_2_T_*x*_ provided additional light-harvesting ability. We believe that high performance electrochemical and photoelectrochemical OER catalysts could be achieved by rational design and combination of dissimilar functional 2D materials.

## Conflicts of interest

There are no conflicts to declare.

## Supplementary Material

RA-008-C8RA02349B-s001
