# Supplementary material for: In situ hybridization of an MXene/TiO2/NiFeCo-layered double hydroxide composite for electrochemical and photoelectrochemical oxygen evolution
Source: RSC Adv. 2018 Jun 5;8(37):20576–84. doi: 10.1039/c8ra02349b (PMC9080836; doi:10.1039/c8ra02349b)
Supplement: RA-008-C8RA02349B-s001 [file RA-008-C8RA02349B-s001.pdf]

Electronic Supplementary Material (ESI) for RSC Advances.
This journal is © The Royal Society of Chemistry 2018

Electronic Supplementary Information (ESI)

In-situ hybridization of MXene/TiO_2/NiFeCo-Layered Double Hydroxide Composite for Electrochemical and Photoelectrochemical Oxygen Evolution

Ningxian Hao,[a] Yang Wei,[a] Jialiang Wang,[a] Zhiwei Wang,[a] Zhaohua Zhu,[a] Shulin Zhao,[b] Min Han[b] and Xiao Huang*[a]

[a] Institute of Advanced Materials (IAM), Nanjing Tech University (Nanjing Tech), 30 South Puzhu Road, Nanjing 211816, P. R .China.

[b] Jiangsu Key Laboratory of Biofunctional Materials School of Chemistry and Materials Science, Nanjing Normal University, 1 Wenyuan Road , Nanjing 210023, P. R. China.

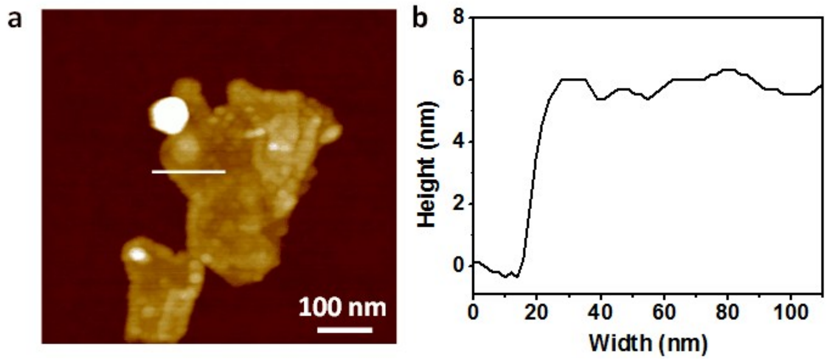

Fig. S1 (a) AFM image and (b) step analysis of typical $Ti_3C_2T_x$ nanosheets.

Fig. S1 showed a typical AFM image of exfoliated nanosheets. It can be seen that one of the nanosheets is about 6 nm in thickness, indicating that it contains 5-6 layers. The surfaces of the nanosheets are not smooth, suggesting that surface oxidation may have occurred.

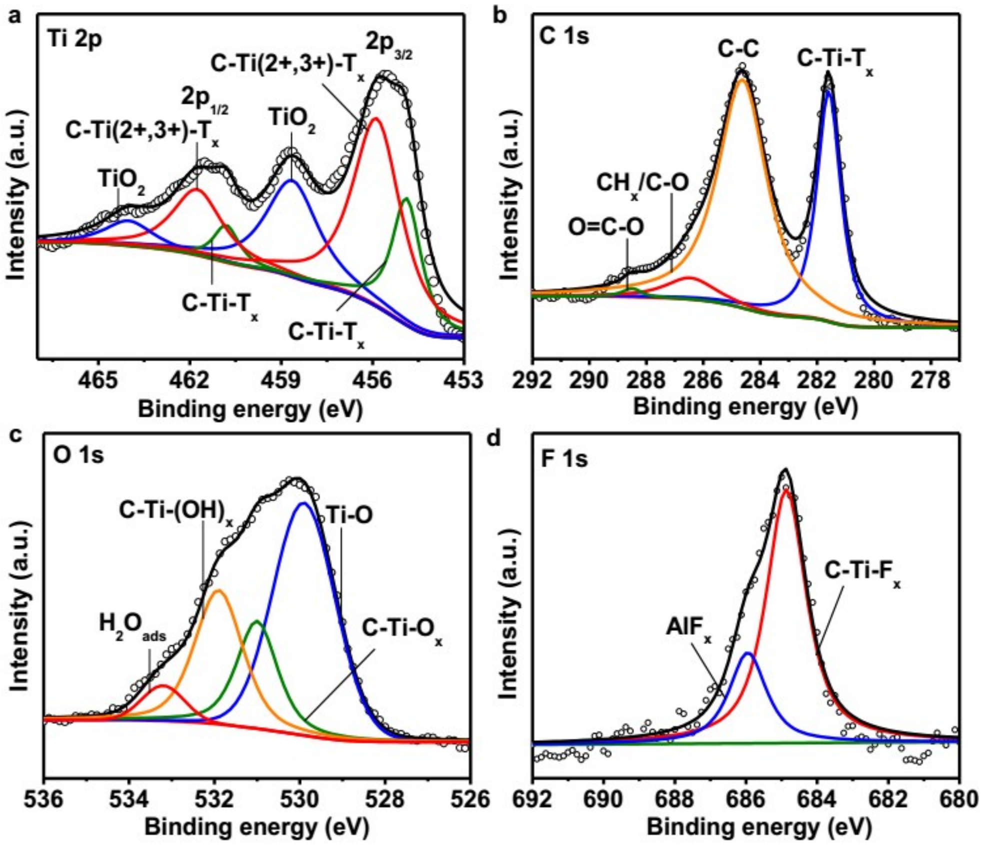

Fig. S2 XPS (a) Ti 2p, (b) C 1s, (c) O 1s and (d) F 1s spectra of as-exfoliated $Ti_3C_2T_x$ nanosheets.

In Fig. S1a, the high resolution Ti 2p spectrum can be deconvoluted to give three sets of doublet peaks. The doublets at 454.9 eV/460.8 eV and 455.9 eV/461.8eV can be attributed to the C−Ti−T_x and the C−Ti(2+, 3+)−T_x (T is O, OH or F) species, respectively.[1,2] The doublet at 458.6 eV/464 eV can be attributed to TiO_2.[1,2] The peak at 281.6 eV in the C 1s spectrum can be assigned to C−Ti−T_x (T is O, OH or F), next to which, the peaks at 284.6 eV, 286.5 eV and 288.5 eV can be assigned to the C−C, CH_x/C−O and O=C−O species, respectively.[2,3] The O 1s spectrum was fitted by components corresponding to surface adsorbed H_2O_{ads} (533.2 eV), C−Ti−$(OH)_x$ (531.9 eV), C−Ti−O_x (531 eV) and Ti−O (529.9 eV) species.[4] For the F 1s spectrum, the peaks at 684.9 eV and 686 eV can be assigned to C−Ti−F_x and AlF_x, respectively.[2]

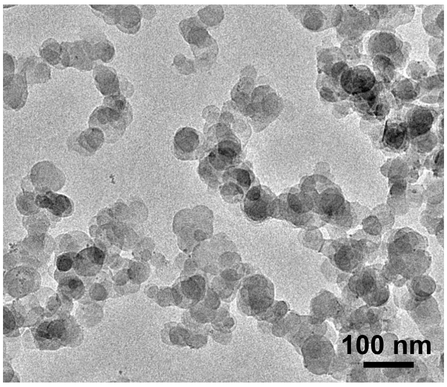

Fig. S3 TEM image of LDH nanoplates.

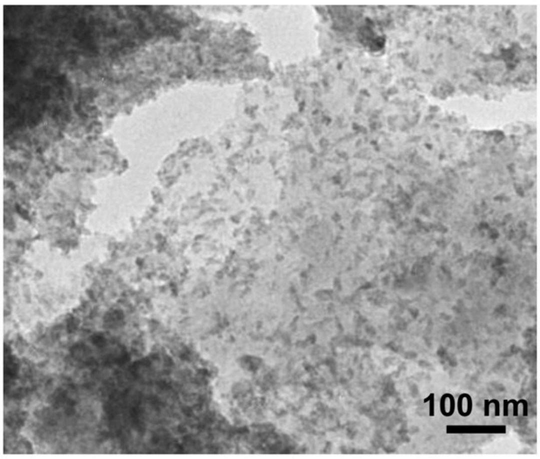

Fig. S4 TEM image of $Ti_3C_2T_x/TiO_2$ obtained by a solvothermal reaction without the presence of LDH precursors.

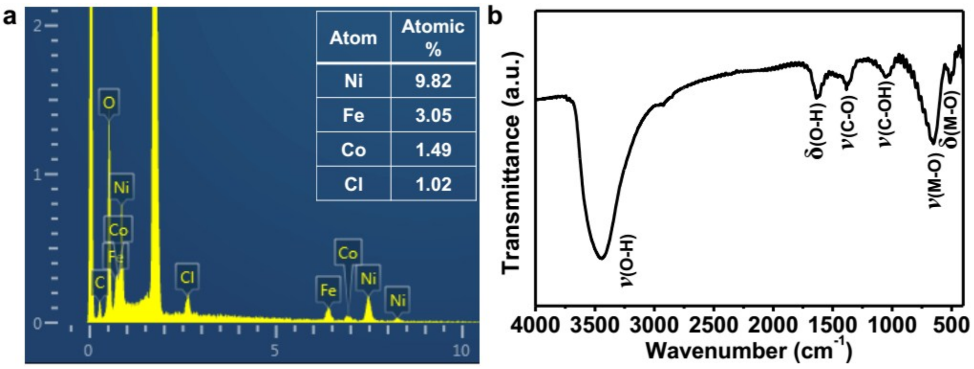

Fig. S5 (a) EDX spectrum and (b) FT-IR spectrum of LDH nanoplates.

In the FT-IR spectrum in (b), the strong peak at ~3439 cm^{-1} corresponds to a combination of the stretching vibration of hydroxide groups and water molecules.[5,6] The peak at ~1634 cm^{-1} belongs to the bending vibrations of water molecules,[5,6] while the band observed in 1045 cm^{-1} is mainly associated with the stretching vibration of C−OH.[8,9] These suggest that a certain amount of residual hydroxyl groups existed in the system together with water molecules. The peak at ~1377 cm^{-1} can be assigned to the ν3 vibration of CO_3^{2-},[5-7] indicating that CO_3^{2-} may exist as the anions in the LDH nanoplates. The peaks at ~656 and ~514 cm^{-1} are related to the metal-oxygen (M−O) lattice vibrations of LDHs.[6,8]

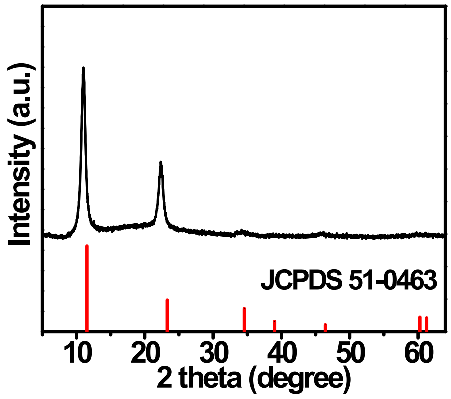

Fig. S6 XRD pattern of LDH nanoplates.

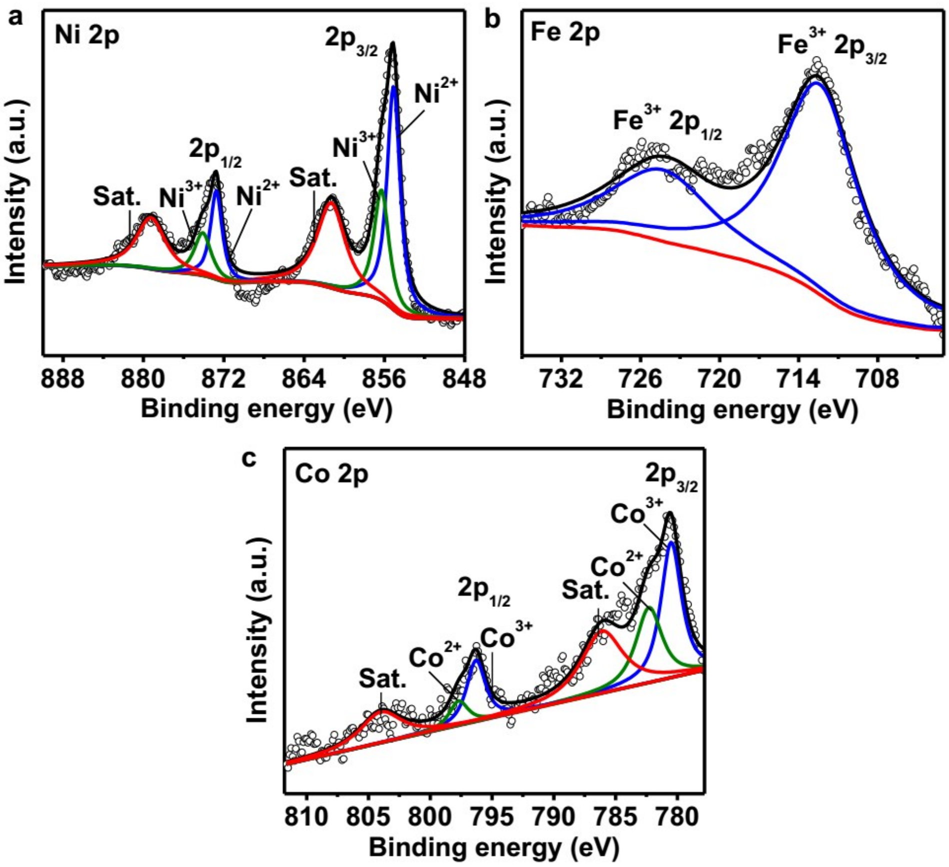

Fig. S7 XPS (a) Ni 2p, (b) Fe 2p and (c) Co 2p spectra of LDH nanoplates.

The Ni 2p spectrum shows two sets of doublet for Ni^{2+} (855.1 eV and 872.7 eV) and Ni^{3+} (856.2 eV and 874 eV), along with two satellite peaks at 861.3 eV and 879.2 eV, respectively.[10-12] The signals at the binding energy of 712.4 eV and 724.3 eV were corresponding to Fe $2p_{3/2}$ and Fe $2p_{1/2}$ bands of Fe(3+).[13,14] The Co 2p spectrum shows two sets of doublet for Co^{2+} (782.3 eV and 797.7 eV) and Co^{3+} (780. 5 eV and 796.3 eV), along with two satellite peaks at 786.1 eV and 804 eV, respectively.[10,12]

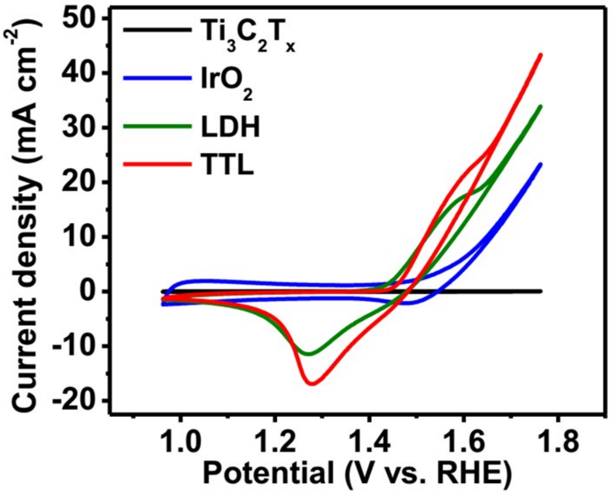

Fig. S8 Cyclic voltammetry (CV) curves of $Ti_3C_2T_x$, LDH, IrO_2 and TTL composite.

The electrocatalytic activity was examined in O_2-saturated 0.1 M KOH by cycling at a potential range of 0.96-1.76 V (*vs.* RHE) at a scan rate of 100 mV s^{-1}. For the NiFeCo-LDH and TTL modified electrodes, the redox peaks at ~1.2-1.6 V (*vs.* RHE) could be assigned to the $Ni^{2+/3+}$ to $Ni^{3+/4+}$ and $Co^{2+/3+}$ to $Co^{3+/4+}$ redox couples.[15,16] In contrast, there were no obvious redox peaks for $Ti_3C_2T_x$.

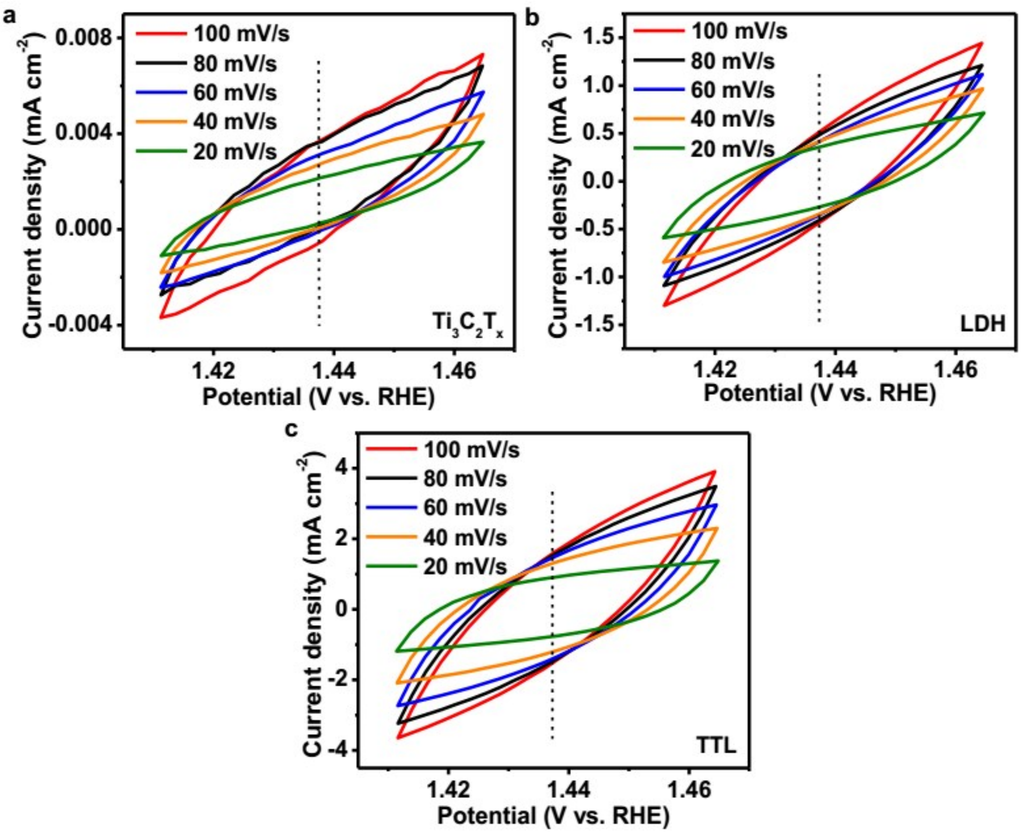

Fig. S9 CV curves of (a) $Ti_3C_2T_x$ nanosheets, (b) NiFeCo-LDH nanoplates and (c) TTL composite at different scan rates of 20, 40, 60, 80, 100 mV s^{-1} in a potential range of 1.411-1.464 V (*vs.* RHE) without apparent Faradic processes.

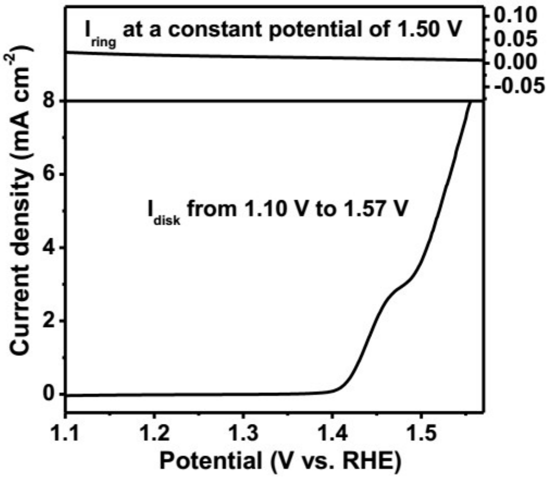

Fig. S10 RRDE measurement of TTL-based electrode conducted in O_2-saturated 0.1 M KOH solution at a rotation speed of 1,600 rpm.

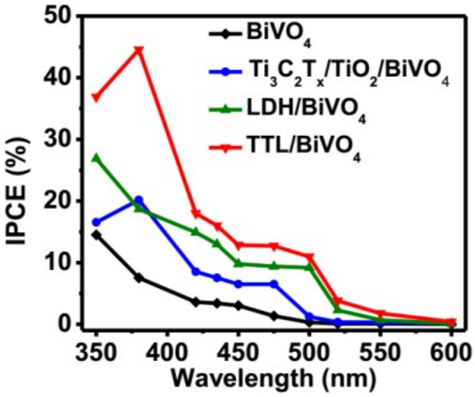

Fig. S11 IPCE measured at 1.23 V *vs*. RHE under monochromatic light irradiation.

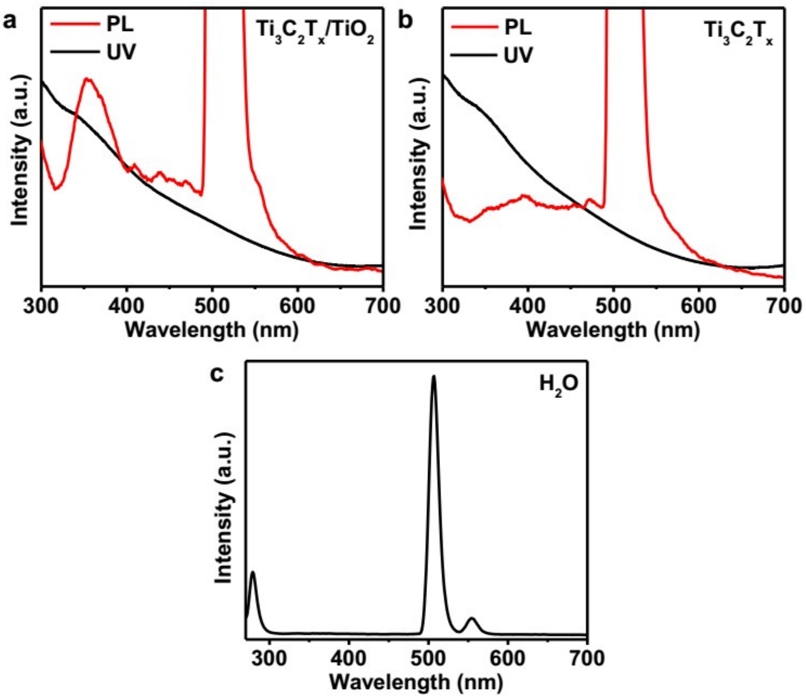

Fig. S12 Normalized UV-vis absorption spectrum and photoluminescence spectrum of (a) $Ti_3C_2T_x/TiO_2$ and (b) $Ti_3C_2T_x$ at $\lambda_{excitation}$ of 250 nm. (c) Photoluminescence spectrum of H_2O ($\lambda_{excitation}$ = 250 nm), indicating that the peaks at about 280 nm and 505 nm are system peaks.

Table S1. Weight content of Ti, Co, Fe and Ni in TTL obtained from the ICP-OES.

Element	Ti	Ni	Fe	Co
**Weight%**	10.7	30.8	10.5	5

Table S2. Atomic percentage of TiO_2 and C-Ti-T_x species based on XPS Ti 2p spectrum of TTL.

Species	Peak area	Atomic%
**TiO_2**	7900	95.4
**C-Ti-T_x**	378.6	4.6

Table S3. Weight content of various components in TTL.

Component	TiO_2	$Ti_3C_2T_x$	NiFeCo-LDH
**Weight%**	17	1.7	81.3

The values obtained in **Table S3** were calculated based on the weight percentage of the various metals, including Ti, Co, Fe and Ni in TTL in **Table S1**, and the atomic percentage of Ti species in TTL shown in **Table S2.**

Table S4. Performance comparison among recently reported OER catalysts (in 0.1 M KOH electrolyte on GC electrode)

Catalyst	Onset potential (V *vs.* RHE)	Tafel slope (mV dec^{-1})	Potential at 10 mA cm^{-2} (V *vs.* RHE)	Catalyst loading (mg cm^{-2})	Reference
**TTL**	1.47	98.4	1.55	0.21	This work
**IrO_2/C**	1.50	N.A.	1.60	0.2	1
**N-doped G/CNT**	1.45	83	1.63	0.25	17
**Pd@PdO–Co_3O_4**	1.40	70	1.54	0.2	18
**Ni_2CoFe-LDH+GO**	1.47	74.5	1.52	0.168	14
**NiFe@NC**	1.55	56	1.58	0.2	19
**$CoFe_{35}$ LDH**	N.A.	49	1.58	0.25	20
**Ti_3C_2-CoBDC**	1.51	48.2	1.64	0.21	3
**Ni-NiO/N-rGO**	~1.36	43	1.47	0.21	21
**Fe_3Co_2-MOF**	1.46	43	1.51	0.22	22
**Fe_1Co_1-ONS**	N.A.	36.8	1.53	0.36	23
**Mn_3O_4/ $CoSe_2$**	N.A.	49	1.68	0.2	24
**Mn_xO_y-N-doped carbon**	1.55	82.6	1.68	0.21	25
**g-C_3N_4-CN**	1.53	83	1.60	0.2	1
**N, S-CN**	N.A.	59	~1.65	0.08	26
**Ni_2CoFe-LDH/N-GO**	1.41	56.8	1.51	0.17	27
**$Ni_{1-x}Fe_x$OOH**	N.A.	55	1.55	N.A.	28
**NiFe-LDH/CNT**	1.50	35	1.54	0.2	29
**CQD/NiFe-LDH**	1.49	30	1.51	0.2	30

Table S5. R_{ct} values for different catalysts to fit the Nyquist plots based on the RC circuit model

**Material**	**$Ti_3C_2T_x$**	**NiFeCo-LDH**	**IrO_2**	**TTL**
**R_{ct} /Ω**	25	40.3	210	33.2

References

(1) L. H. Karlsson, J. Birch, J. Halim, M. W. Barsoum and P. Persson, *Nano Lett.*, 2015, **15**, 4955-4960.

(2) J. Halim, M. R. Lukatskaya, K. M. Cook, J. Lu, C. R. Smith, L. Naslund, S. J. May, L. Hultman, Y. Gogotsi and P. Eklund, *Chem. Mater.*, 2014, **26**, 2374-2381.

(3) L. Zhao, B. Dong, S. Li, L. Zhou, L. Lai, Z. Wang, S. Zhao, M. Han, K. Gao, M. Lu, X. Xie, B. Chen, Z. Liu, X. Wang, H. Zhang, H. Li, J. Liu, H. Zhang, X. Huang and W. Huang, *ACS Nano*, 2017, **11**, 5800-5807.

(4) Y. C. G. Kwan, G. M. Ng and C. H. A. Huan, *Thin Solid Films*, 2015, **590**, 40-48.

(5) C. J. Wang and D. O'Hare, *J. Mater. Chem.*, 2012, **22**, 21125-21130.

(6) Z. Liu, R. Ma, M. Osada, N. Iyi, Y. Ebina, K. Takada and T. Sasaki, *J. Am. Chem. Soc.*, 2006, **128**, 4872-4880.

(7) M. Wei, S. Shi, J. Wang, Y. Li and X. Duan, *J. Solid State Chem.*, 2004, **177**, 2534-2541.

(8) J. Li, Q. Fan, Y. Wu, X. Wang, C. Chen, Z. Tang and X. Wang, *J. Mater. Chem. A*, 2016, **4**, 1737-1746.

(9) X. Sun and Y. Li, *Angew. Chem. Int. Ed.*, 2004, **43**, 597-601.

(10) J. Jiang, A. Zhang, L. Li and L. Ai, *J. Power Sources*, 2015, **278**, 445-451.

(11) Q. Yang, T. Li, Z. Lu, X. Sun and J. Liu, *Nanoscale*, 2014, **6**, 11789-11794.

(12) R. Zou, K. Xu, T. Wang, G. He, Q. Liu, X. Liu, Z. Zhang and J. Hu, *J. Mater. Chem. A*, 2013, **1**, 8560-8566.

(13) L. Zhou, X. Huang, H. Chen, P. Jin, G. Li and X. Zou, *Dalton Trans.*, 2015, **44**, 11592-11600.

(14) L. Qian, Z. Lu, T. Xu, X. Wu, Y. Tian, Y. Li, Z. Huo, X. Sun and X. Duan, *Adv. Energy Mater.*, 2015, **5**, 1500245.

(15) S. Klaus, M. W. Louie, L. Trotochaud and A. T. Bell, *J. Phys. Chem. C*, 2015, **119**, 18303-18316.

(16) Y. P. Zhu, T. Ma, M. Jaroniec and S. Z. Qiao, *Angew. Chem. Int. Ed.*, 2017, **56**, 1324-1328.

(17) G. L. Tian, M. Q. Zhao, D. Yu, X. Y. Kong, J. Q. Huang, Q. Zhang and F. Wei, *Small*, 2014, **10**, 2251-2259.

(18) H. C. Li, Y. J. Zhang, X. Hu, W. J. Liu, J. J. Chen and H. Q. Yu, *Adv. Energy Mater.*, 1702734.

(19) Z. Zhang, Y. Qin, M. Dou, J. Ji and F. Wang, *Nano Energy*, 2016, **30**, 426-433.

(20) F. Yang, K. Sliozberg, I. Sinev, H. Antoni, A. Bähr, K. Ollegott, W. Xia, J. Masa, W. Grünert, B. R. Cuenya, W. Schuhmann and M. Muhler, *ChemSusChem*, 2017, **10**, 156-165.

(21) X. Liu, W. Liu, M. Ko, M. Park, M. G. Kim, P. Oh, S. Chae, S. Park, A. Casimir, G. Wu and J. Cho, *Adv. Funct. Mater.*, 2015, **25**, 5799-5808.

(22) J. Q. Shen, P. Q. Liao, D. D. Zhou, C. T. He, J. X. Wu, W. X. Zhang, J. P. Zhang and X. M. Chen, *J. Am. Chem. Soc.*, 2017, **139**, 1778-1781.

(23) L. Zhuang, L. Ge, Y. Yang, M. Li, Y. Jia, X. Yao and Z. Zhu, *Adv. Mater.*, 2017, **29**, 1606793.

(24) M. Gao, Y. Xu, J. Jiang, Y. Zheng and S. Yu, *J. Am. Chem. Soc.*, 2012, **134**, 2930-2933.

(25) J. Masa, W. Xia, I. Sinev, A. Zhao, Z. Sun, S. Grutzke, P. Weide, M. Muhler and W. Schuhmann, *Angew. Chem. Int. Ed.*, 2014, **53**, 8508-8512.

(26) K. Qu, Y. Zheng, S. Dai and S. Z. Qiao, *Nano Energy*, 2016, **19**, 373-381.

(27) D. Zhou, Z. Cai, X. Lei, W. Tian, Y. Bi, Y. Jia, N. Han, T. Gao, Q. Zhang, Y. Kuang, J. Pan, X. Sun and X. Duan, *Adv. Energy Mater.*, 2017, 1701905.

(28) S. Klaus, M. W. Louie, L. Trotochaud and A. T. Bell, *J. Phys. Chem. C*, 2015, **119**, 18303-18316.

(29) M. Gong, Y. Li, H. Wang, Y. Liang, J. Z. Wu, J. Zhou, J. Wang, T. Regier, F. Wei and H. Dai, *J. Am. Chem. Soc.*, 2013, **135**, 8452-8455.

(30) D. Tang, J. Liu, X. Wu, R. Liu, X. Han, Y. Han, H. Huang, Y. Liu and Z. Kang, *ACS Appl. Mater. Inter.*, 2014, **6**, 7918-7925.
